# Tumor necrosis factor alpha-dependent aggrecan cleavage and release of glycosaminoglycans in the meniscus is mediated by nitrous oxide-independent aggrecanase activity in vitro

**DOI:** 10.1186/ar2813

**Published:** 2009-09-24

**Authors:** Henning Voigt, Angelika K Lemke, Rolf Mentlein, Michael Schünke, Bodo Kurz

**Affiliations:** 1 Institute of Anatomy, Christian-Albrechts-University Kiel, Olshausenstr. 40, Kiel, 24098, Germany

## Abstract

**Introduction:**

Little is known about factors that induce meniscus damage. Since joint inflammation appears to be a causative factor for meniscal destruction, we investigated the influence of tumor necrosis factor (TNFα) on glycosaminoglycan (GAG) release and aggrecan cleavage in an *in vitro *model.

**Methods:**

Meniscal explant disks (3 mm diameter × 1 mm thickness) were isolated from 2-year-old cattle. After 3 days of TNFα-treatment GAG release (DMMB assay), biosynthetic activity (sulfate incorporation), nitric oxide (NO) production (Griess assay), gene expression of matrix-degrading enzymes (quantitative RT-PCR, zymography), and immunostaining of the aggrecan fragment NITEGE were determined.

**Results:**

TNFα induced release of GAG as well as production of NO in a dose-dependent manner, while sulfate incorporation was decreased. TNFα increased matrix metalloproteinase (MMP)-3 and a disintegrin and metalloproteinase with thrombospondin motifs (ADAMTS)-4 mRNA expression, whereas collagen type I was decreased, and aggrecan, collagen type II as well as MMP-1, -2, -13 and ADAMTS-5 were variably affected. Zymography also showed a TNFα-dependent increase in MMP-3 expression, but pre-dominantly in the pro-form. TNFα-dependent formation of the aggrecanase-specific aggrecan neoepitope NITEGE was induced. Tissue inhibitor of metalloproteinases (TIMP)-3, but not TIMP-1 or -2 inhibited TNFα-dependent GAG release and NITEGE production, whereas inhibition of TNFα-dependent NO generation with the NO-synthetase inhibitor L-NMMA failed to inhibit GAG release and NITEGE production.

**Conclusions:**

Our study shows that aggrecanase activity (a) is responsible for early TNFα-dependent aggrecan cleavage and GAG release in the meniscus and (b) might be involved in meniscal degeneration. Additionally, the meniscus is a TNFα-dependent source for MMP-3. However, the TNFα-dependent NO production seems not to be involved in release of proteoglycans under the given circumstances.

## Introduction

Meniscal function and integrity are crucial for a healthy knee joint, because damage to the tissue subsequently leads to articular cartilage destruction and further degenerative diseases such as osteoarthritis (OA) [1-3]. In order to restore the meniscal function it is important to understand the pathomechanisms of meniscal destruction.

Increased levels of nitric oxide (NO) and pro-inflammatory cytokines, such as TNFα and IL-1, have been found in the synovial fluid and tissues of inflamed joints [4,5]. It is also well established that cytokines can be involved in cartilage tissue or proteoglycan degradation [6]. It has recently been shown in a serum-containing porcine *in vitro *model that these cytokines are able to inhibit the intrinsic meniscal repair response [7,8], and part of this effect has been found to be mediated by the activation of matrix metalloproteinases (MMPs) [9,10]. The patterns of enzyme expression during experimental OA suggest that there are similarities in the involvement of MMPs and aggrecanases in the degradation of menisci and articular cartilage [11]. It is therefore suggested that members of the MMPs as well as the a disintegrin and metalloproteinase with thrombospondin motifs (ADAMTS) family, such as ADAMTS-4 (aggrecanase-1) and ADAMTS-5 (aggrecanase-2), must also be involved in cytokine-dependent degradation of proteoglycans in the meniscus. Meniscal expression and biomechanical regulation of all these enzymes has recently been shown in a porcine tissue explant model [12]. Aggrecanases are known to be responsible for aggrecan degradation in articular cartilage in diseases such as OA and rheumatoid arthritis (RA) [13], and cleave the aggrecan core protein at several specific sites; one is between Glu^373 ^and Ala^374 ^which generates the G1-NITEGE fragment [14,15].

It has been shown in many studies that meniscal tissue can produce NO during experimental OA [4], or after partial meniscectomy [16], mechanical stimulation [17-19], or cytokine treatment with IL-1 or TNFα [20-22]. However, the mechanisms of endogenous NO involvement in meniscal degeneration still remain unclear. It is associated with cartilage tissue destruction [19,23], but was also found to protect from IL-1-mediated proteoglycan degradation [21].

In order to investigate the influence of TNFα on the meniscus we present a bovine *in vitro *model that allows the isolation of meniscal tissue explants of defined geometry and anatomical location. Using this model we study the effect of TNFα on glycosaminoglycan (GAG) release, biosynthetic activity, NO production, aggrecan fragmentation (because aggrecan has been described as one of the major proteoglycans in the meniscus [24]), and gene expression of matrix molecules, MMPs and aggrecanases in the meniscus. We demonstrate that within three days of incubation there is a TNFα-dependent up-regulation of MMP-3 and ADAMTS-4 expression, as well as aggrecanase activity. The latter induces GAG release, cleaves aggrecan at the NITEGE site and is independent of the TNFα-induced NO production.

## Materials and methods

### Isolation and culturing of meniscal explant disks

Meniscal explant disks were isolated from bovine menisci (from 16 to 24 month old cattle), procured from a local abattoir with authorization from the relevant meat inspectors. This study does not involve human subjects, human tissue or experimentation of animals. Up to four full thickness tissue cylinders (10 mm in diameter) per meniscus were punched perpendicular to the meniscus bottom surface. Tissue disks 1 mm in thickness were sliced including the original meniscal surface using a sterile scalpel blade, and four to five smaller explant disks (3 mm in diameter × 1 mm thick) were isolated using a biopsy punch (HEBUmedical, Tuttlingen, Germany) and cultured in DMEM (supplemented with 100 U/ml penicillin G, 100 μg/ml streptomycin, and 0.25 μg/ml amphotericin B; Sigma-Aldrich, St. Louis, MO, USA) in a 37°C, 5% CO_2 _environment after measurement of wet weight. The total of up to 60 explants per animal (2 knee joints including medial/lateral menisci) were randomised among the different experimental groups matched by their anatomical location for every single experiment and cultured in the absence or presence of varying concentrations of recombinant human TNFα (R & D Systems, Minneapolis, MN, USA). In most of the experiments a concentration of 100 ng TNFα/ml was used. Three explant disks per well of a 24-well plate were cultured in 1 ml medium. After three days of culture the medium and explants were used for measurements. For inhibitory studies different tissue inhibitor of metalloproteinases (TIMPs; R & D Systems, Minneapolis, MN, USA) and the NO synthetase inhibitor L-NMMA were used. For these investigations only one meniscal explant per well was cultured for three days in 200 μl medium in 96-well plates.

### Immunohistochemistry

The meniscal explants were fixed overnight in 4% paraformaldehyde and embedded in paraffin. Serial sections (7 μm) were cut sagittally through the entire thickness of the explant disks, immobilised on glass slides, and deparaffinised. After incubation for 2.5 minutes in a digester at 100°C (in 0.01 M citric acid, pH 6.0), they were incubated overnight at 4°C with the primary antibody (anti-NITEGE; 1:50 dilution in 1% BSA; ABR Affinity BioReagents, Golden, CO, USA), rinsed in Tris-NaCl three times for five minutes and incubated with the secondary antibody AlexaFluor 488 goat anti-rabbit IgG (1:500; Invitrogen, Carlsbad, CA, USA) for one hour at room temperature. After further washing, the sections were labeled for nuclear staining with bisbenzimide (Sigma, St. Louis, MO, USA), mounted with fluorescence mounting medium (Dako, Glostrup, Denmark), and visualised using the Apotome (ZEISS, Jena, Germany) fluorescence microscope.

### Measurement of biosynthetic activity, glycosaminoglycans and nitric oxide production

For radiolabel incorporation the meniscal explants were placed in fresh culture medium containing 10 μCi/ml [^35^SO_4_]-sulfate (Amersham Pharmacia, GE Healthcare Europe GmbH, Munich, Germany) for six to eight hours at 37°C under free-swelling conditions right after cytokine treatment. Afterwards, the explants were washed in PBS containing 0.5 mM proline and digested overnight in 1 ml of papain solution (0.125 mg/ml (2.125 U/ml, Sigma, St. Louis, MO, USA), 0.1 M Na_2_HPO_4_, 0.01 M Na-EDTA, 0.01 M L-cysteine, pH 6.5) at 65°C. A 200 μl aliquot of each sample were added to 2 ml scintillation fluid (Opti Phase Hi Safe 3, Perkin Elmer, Waltham, MA, USA) and measured using a Beckmann scintillation counter (Wallac 1904. Turku, Finland). Counts were expressed in cpm/mg wet weight and normalised to the radiolabel incorporation of untreated control tissue, which was set to 100%.

For measurement of GAG release or content the media were collected after cytokine treatment or the papain-digested explants were used (see above), and GAG content was determined by DMMB dye assay photometrically at a wavelength of 520 nm (Photometer Ultraspec II, Biochrom, Cambridge, UK) using shark chondroitin-sulfate as standard. Values were presented as μg GAG per mg wet weight of the explants.

Generation of NO was determined by measuring nitrite accumulation in culture supernatants using Griess reagent (1% sulfanilamide and 0.1% N-(1-naphtyl)-ethylene diamine-dihydro-chloride in 5% H_3_PO_4_, Sigma-Aldrich, St. Louis, MO, USA). A 100 μl aliquot of each sample and 100 μl Griess reagent were mixed and incubated for five minutes, and the absorption was determined in an automated plate reader (SLT Reader 340 ATTC, SLT-Labinstruments, Achterwehr, Germany) at 540 nm. Sodium nitrite (NaNO_2_, Merck, Darmstadt, Germany) was used to generate a standard curve for quantification.

### Quantitative RT-PCR

After three days of incubation, quantitative real-time RT-PCR was performed using glyceraldehyde-3-phosphate dehydrogenase (GAPDH) as reference gene to determine gene expression levels. Meniscal explants (approximately 100 mg from each group) were frozen immediately in liquid nitrogen. Total RNA was extracted after pulverisation of the tissue using the TRIZOL reagent (1 ml/100 mg wet weight tissue; Invitrogen, Carlsbad, CA, USA) followed by extraction with chloroform and isopropanol precipitation. The concentration of extracted RNA was quantified spectro-photometrically at OD_260_/OD_280 _nm. Before real-time RT-PCR was performed using the Qiagen QuantiTect SYBR^® ^Green RT-PCR Kit (Qiagen, Hilden, Germany) according to the manufacturer's instructions the extracted RNA was digested with DNase (65°C for 10 minutes; Promega, Madison, WI, USA) to remove any traces of DNA. Bovine primers were designed using Primer3 Software [25] and used at a concentration of 0.5 μM (Table 1). Conditions for real-time RT-PCR were as specified by manufacturer's description: reverse transcription 30 minutes at 50°C; PCR initial activation step 15 minutes at 95°C; denaturation 15 seconds at 94°C; annealing 30 seconds at 60°C; extension 30 seconds at 72°C; optional: data acquisition 30 seconds at melting temperature 70 to 78°C. Differences of mRNA levels between control and stimulated samples were calculated using the ΔΔC_T_-method. ΔC_T _represents the difference between the C_T _(cycle of threshold) of a target gene and the reference gene (GAPDH). The ΔΔC_T _value is calculated as the difference between ΔC_T _from the stimulated samples and the control.

**Table 1:** List of primers used for real time RT-PCR

Target	Sequence (5' to 3')	Product size
GAPDH *S*	ATC AAG AAG GTG GTG AAG CAG G	101 bp
GAPDH *AS*	TGA GTG TCG CTG TTG AAG TCG	
18sRNA *S*	TCG AGG CCC TGT AAT TGG AA	104 bp
18sRNA *AS*	GCT ATT GGA GCT GGA ATT ACC G	
Aggrecan *S*	CCT GAA CGA CAA GAC CAT CGA	101 bp
Aggrecan *AS*	TGG CAA AGA AGT TGT CAG GCT	
Collagen type I *S*	AAT TCC AAG GCC AAG AAG CAT G	102 bp
Collagen type I *AS*	GGT AGC CAT TTC CTT GGT GGT T	
Collagen type II *S*	AAG AAG GCT CTG CTC ATC CAG G	124 bp
Collagen type II *AS*	TAG TCT TGC CCC ACT TAC CGG T	
MMP-1 *S*	GGA CTG TCC GGA ATG AGG ATC T	91 bp
MMP-1 *AS*	TTG GAA TGC TCA AGG CCC A	
MMP-2 *S*	GTA CGG GAA TGC TGA CGG GGA ATA	93 bp
MMP-2 *AS*	CCA TCG CTG CGG CCT GTG TCT GT	
MMP-3 *S*	CAC TCA ACC GAA CGT GAA GCT	109 bp
MMP-3 *AS*	CGT ACA GGA ACT GAA TGC CGT	
MMP-13 *S*	TCT TGT TGC TGC CCA TGA GT	101 bp
MMP-13 *AS*	GGC TTT TGC CAG TGT AGG TGT A	
ADAMTS-4 *S*	GCG CCC GCT TCA TCA CTG	101 bp
ADAMTS-4 *AS*	TTG CCG GGG AAG GTC ACG	
ADAMTS-5 *S*	AAG CTG CCG GCC GTG GAA GGA A	196 bp
ADAMTS-5 *AS*	TGG GTT ATT GCA GTG GCG GTA GG	

ADAMTS = a disintegrin and metalloproteinase with thrombospondin motifs; AS = antisense; bp = base pairs; GAPDH = glyceraldehyde-3-phosphate dehydrogenase; MMP = matrix-metalloproteinase; S = sense.

### Zymography

Protein levels of MMPs were assayed in conditioned media by gelatin and casein zymography. Equal volumes of medium samples and loading buffer (2 mM EDTA, 2% (w/v) SDS, 0.02% (w/v) bromophenol blue, 20 mM Tris-HCl, pH 8.0) were mixed, subjected to electrophoresis using 0.1% (w/v) gelatin and 0.2% (w/v) casein as substrate in 4.5 to 15% gradient SDS-PAGE, washed in 2.5% (v/v) Triton X-100, rinsed in distilled water and incubated for 16 hours at 37°C in 50 mM Tris-HCL (pH 8.5) containing 5 mM CaCl_2_. Gels were stained with 0.1% (w/v) Coomassie brilliant blue R250 (Serva, Heidelberg, Germany) and destained with 10% (v/v) acetic/50% (v/v) methanol and with 10% (v/v) acetic acid/10% (v/v) methanol. MMPs were identified by molecular weight and substrate specificity as clear bands against a blue background of undigested substrate. Additionally, samples were incubated with 1 mM 4-aminophenylmercuric acetate (APMA; Sigma-Aldrich, St. Louis, MO, USA) for three hours at 37°C to activate MMP-pro-forms prior to loading.

### Statistics

Quantitative data are presented as mean ± standard error of the mean, n represents the number of independent experiments. Statistical analysis of data was made using a one-way analysis of variance (ANOVA) indicating significant differences, and comparisons among the various experimental groups were made using the two-tailed Student's t-test. Differences were considered significant if *P *≤ 0.05.

## Results

### TNFα-dependent GAG release

**Figure 1:**
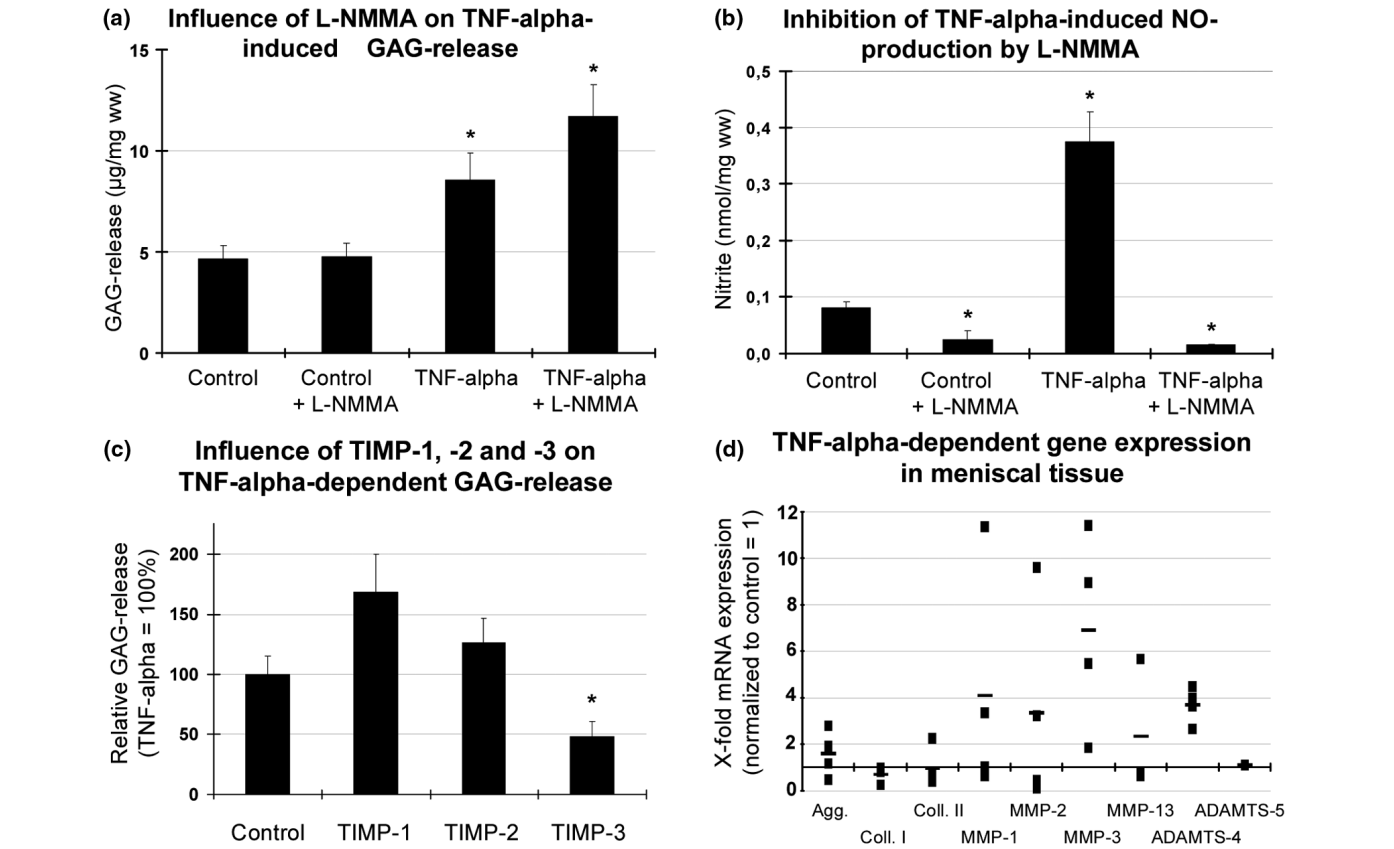
Influence of a three-day incubation with TNFα (100 ng/ml), the NO synthetase inhibitor L-NMMA (1 mM), and the TIMPs (0.1 μM) on the GAG-release, NO production and gene expression level of bovine meniscal tissue explants. **(a) **Cumulative glycosaminoglycan (GAG) release (n = 6). **(b) **Cumulative nitric oxide (NO) production, measured by photometrical detection of nitrite accumulation (n = 6). **(c) **Influence of tissue inhibitors of metalloproteinases (TIMPs) on TNFα-dependent GAG release (n = 5). **(d) **TNFα-dependent mRNA levels given as a ratio: the x-fold expression level compared with un-stimulated control tissue (using the ΔΔCT method with GAPDH as reference gene; control = 1). Each dot represents data from an independent experiment, bars indicate the mean from four independent experiments. (a to c) All values are mean ± standard error of the mean. * significantly different from control, *P *< 0.05. ADAMTS = a disintegrin and metalloproteinase with thrombospondin motifs; Agg = aggrecan; Coll I or II = collagen type I or II; MMP = matrix metalloproteinase.

We have established an *in vitro *model for the investigation of bovine meniscal tissue destruction where tissue explant disks (3 mm in diameter and 1 mm thick) were isolated from the meniscal bottom surface (facing the tibial articular cartilage). Mean GAG content of freshly isolated explants was 14.2 ± 0.8 μg/mg wet weight (n = 8). After three days of culture, 4.8 ± 0.3 μg/mg of GAG was released into the media in control explants (normalised to the mean GAG content of fresh explants about one-third of explant GAG is being released during culture). Stimulation with TNFα induced a dose-dependent increase in GAG release: using a concentration of 1 ng/ml caused an additional but non-significant increase in GAG release of approximately 8.8 ± 3.7% compared with control release. With 10 ng TNFα/ml, GAG release increased significantly by 30 ± 12% (n = 11), and 100 ng TNF α/ml (chosen for all subsequent experiments; Figure 1a) increased GAG release significantly by 24 ± 10% (n = 11). In order to distinguish between the release of existing GAG or newly synthesised GAG, radiolabeled sulfate was incorporated after cytokine treatment. TNFα induced a significant reduction in sulfate uptake (controls: 100 ± 12% vs TNFα: 55 ± 11%; n = 4), suggesting that the TNFα-dependent increase in media GAG content must be predominantly the result of an increased matrix degradation, rather than an increased biosynthetic activity.

### TNFα-dependent NO production

TNFα induced a dose-dependent (not shown) and significantly increased production of NO in meniscal explants which increased about four-fold in comparison to the un-stimulated control (Figure 1b). The NO-synthetase inhibitor L-NMMA reduced the basal NO production of the tissue significantly and prevented the TNFα-mediated increase in NO completely.

### Influence of NO synthetase inhibition and TIMPs on TNFα-dependent GAG release

It has been described that proteoglycan degradation in cartilage tissues can be mediated by both the production of NO and the involvement of matrix-degrading enzymes. We therefore studied the influence of the NO-synthetase inhibitor L-NMMA on meniscal tissue. L-NMMA had no significant influence on the basal GAG release and did not reduce the TNFα-induced effect (Figure 1a). There was a slight, but not significant, increase of GAG release instead. In order to support the hypothesis that aggrecanases are involved in TNFα-dependent GAG release, we studied the influence of TIMP-1, -2 and -3. TIMPs are known as specific inhibitors of MMPs, but it has been reported that TIMP-3 has the additional ability to inhibit the aggrecanases ADAMTS-4 and -5 [26,27]. TIMPs did not affect the GAG release in control cultures (not shown). However, the TNFα-induced GAG release was significantly reduced by TIMP-3 by approximately 52% (Figure 1c), whereas TIMP-1 and TIMP-2 showed a trend to increase the TNFα-induced GAG release, although this effect was not significant.

### Expression of matrix molecules and matrix degrading enzymes

To further determine the mechanisms of TNFα-dependent GAG release, the mRNA of meniscal explants was analyzed after a three-day incubation by quantitative RT-PCR. GAPDH had been used as a reference gene, and it had been tested that there is no significant alteration in the C_T _values of GAPDH expression under the influence of TNFα (control: 27.1 ± 1.7 versus TNFα: 27.3 ± 0.9; n = 4 independent experiments). Additionally, GAPDH expression had been tested in relation to another housekeeping gene, 18sRNA: the ratio of GAPDH expression remained unaffected under the influence of TNFα (1.03).

**Figure 2:**
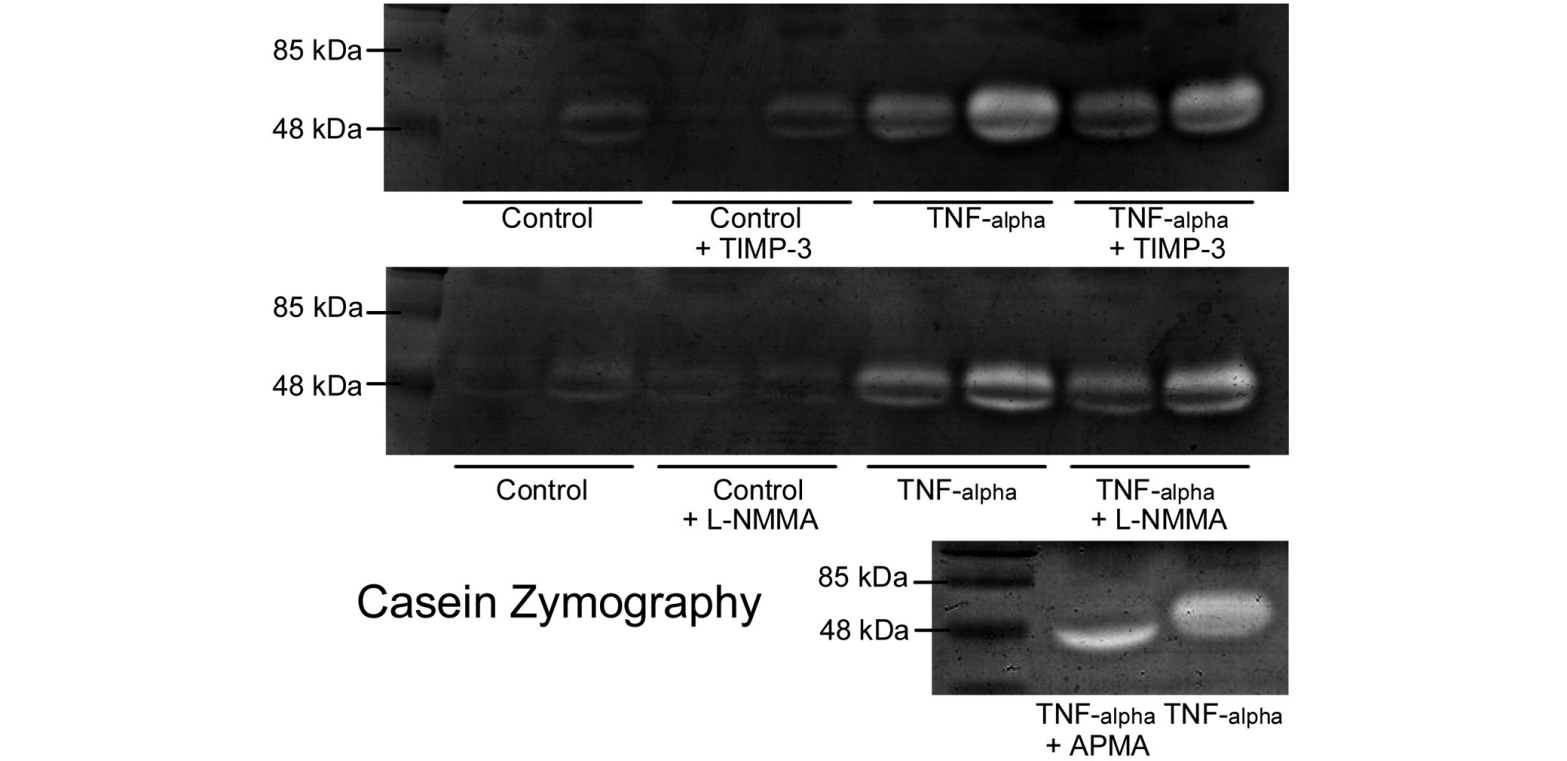
Casein zymograms of culture supernatants after a three day-incubation of meniscal explants under the influence of TNFα, TIMP-3, L-NMMA, or APMA. There are samples from two independent experiments (2 lanes/group) in the upper two zymograms. There is only one major band visible at about 57 kDa (typical size of MMP-3 pro-form [27]) with lower intensity in control cultures and stronger intensity in TNFα-treated samples. TIMP-3 and L-NMMA have no influence on band intensities. The MMP activator APMA (see lower zymogram) reduces the molecular size of the band (45 kDa) and indicates that the enzyme is pre-dominantly expressed as a pro-form. APMA = p-aminophenyl mercuric acetate; L-NMMA = NG-monomethyl-L-arginine.monoacetate; MMP = matrix metalloproteinase; TIMP = tissue inhibitor of metalloproteinases.

The mRNA levels of most of the genes tested were quite variable under the influence of TNFα except for the matrix-degrading enzymes MMP-3 and ADAMTS-4 (see below). Collagen type I mRNA was decreased in all cases (0.75 ± 0.15), while aggrecan and collagen type II as well as MMP-1 and MMP-13 showed both increases and decreases depending on the experiment. ADAMTS-5 was not detectable in some cases or not increased by TNFα. MMP-3 and ADAMTS-4 showed a mean TNFα-dependent 6.9 ± 2.1 and 3.7 ± 0.8-fold increase of mRNA expression (Figure 1d). Comparing delta-C_T_-values (C_TGAPDH _- C_Tgene of interest_) of controls and TNFα-stimulated meniscal explants allows a statistical analysis and showed a significant mean change of about 2.5 ± 0.58 for MMP-3 and 1.86 ± 0.16 for ADAMTS-4, indicating a clear up-regulation of these enzymes in all four independent experiments. The TNFα-dependent MMP-3 expression was also detectable in the supernatants of the cultures by casein zymography (Figure 2). There was only one band detectable in the gels, which was missing or expressed at lower levels in controls, but strong in TNFα-stimulated cultures. This band was not visible in gelatin zymograms (not shown), and had a molecular size of about 57 kDa (typical size for MMP-3, [28]). TIMP-3 as well as L-NMMA had no influence on the band intensity. However, the enzyme activator substance APMA altered the size of the band, indicating that most of the enzyme was expressed as a pro-form [28].

### Aggrecan degradation

**Figure 3:**
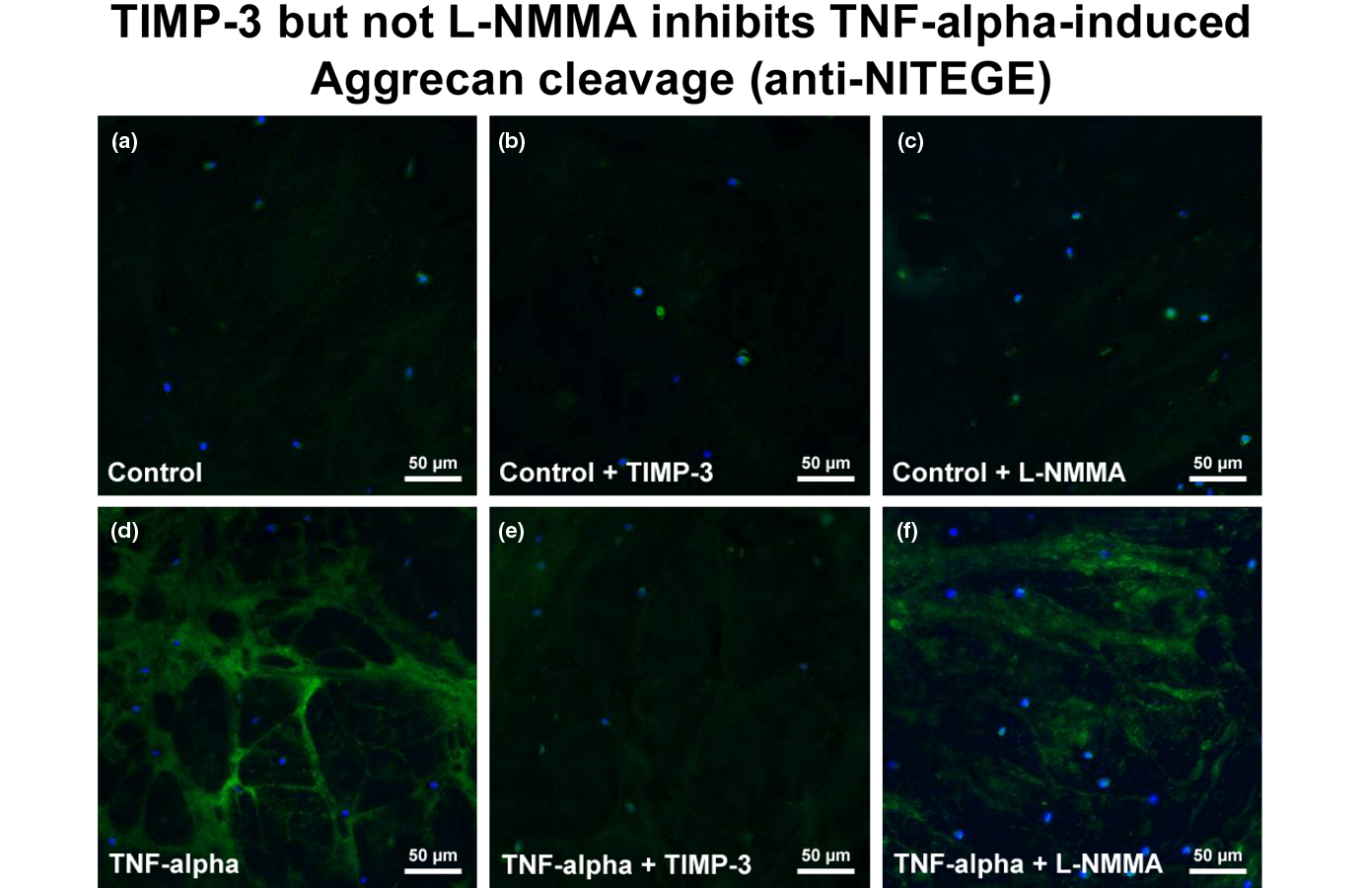
Immunostaining of the aggrecan cleavage product NITEGE in paraffin sections of meniscal explants after three days of incubation with or without TNFα, the protease inhibitor TIMP-3 or the NO synthetase inhibitor L-NMMA. There is an increase in NITEGE-staining (green fluorescence) in **(d) **TNFα-treated samples in comparison to **(a, b, c) **control tissues, and **(e) **TIMP-3 is able to inhibit formation of NITEGE **(f) **in contrast to L-NMMA. Cellular nuclei are counterstained using bisbenzimide (blue fluorescence). L-NMMA = NG-monomethyl-L-arginine.monoacetate; NO = nitric oxide; TIMP = tissue inhibitor of metalloproteinases.

Immunostaining of the aggrecanase activity-specific aggrecan neoepitope NITEGE showed very low signals in control tissue with a clear TNFα-dependent increase in staining in all meniscal tissue areas that could be characterised as fibrous cartilage (Figures 3a and 3d). Co-incubation with the NO-synthetase inhibitor L-NMMA failed to influence the TNFα-dependent NITEGE formation (Figures 3c and 3f), whereas TIMP-3 clearly inhibited this effect (Figures 3b and 3e).

## Discussion

Cartilage catabolism is initiated by proteoglycan degradation followed by that of collagen fibers. Therefore, our study focused on the TNFα-dependent depletion of proteoglycans in a three-day bovine *in vitro *meniscal model [29]. TNFα induced a dose-dependent increase in GAG release supporting data from other investigations on pro-inflammatory cytokines in which IL-1 promoted GAG release in lapine and porcine meniscal tissue [19,21]. TNFα, therefore, appears to be another key factor in meniscal diseases.

To study the mechanisms of TNFα-dependent proteoglycan degradation we investigated the transcription of different matrix-degrading enzymes. One limitation in our study is that aggrecanases had been detected on the mRNA level only; there is no measurement of enzyme proteins, which could help to specify the degradative potencies of enzymes involved in TNFα-dependent proteoglycan degradation. A reason for the missing protein detection is that enzyme levels in the tissue are quite low compared with the large amounts of matrix proteins. We performed immunostainings in tissue sections (not shown), but differences in ADAMTS-4 expression were hard to differentiate, probably due to the fact that immunohistochemistry is not useful for the differentiation of slightly variable expression levels. We therefore mainly focus on the effect of inhibitors such as TIMPs or NO synthetase inhibitor (L-NMMA), and the cleavage products of aggrecan (NITEGE), which both suggest that aggrecanases must be involved in the early TNFα-dependent aggrecan degradation and GAG release in the meniscus (see below).

Increased concentrations of MMPs have been found in animal models of OA, in osteoarthritic human articular cartilage and in the synovial fluid of RA and OA patients [11,30-33], but only little is known about the extent to which the meniscus might be involved in the production of these enzymes. We demonstrate that the meniscus can be an additional source for MMP-3 production, especially under the influence of TNFα. Wilson and colleagues [34] emphasise the importance of MMP activity in meniscal proteoglycan degradation after a 12-day incubation of bovine meniscal tissue from one to two-weekold calves with 20 ng/ml IL-1 and different enzyme inhibitors, but the authors do not specify the kind of MMPs. Additionally, Wilusz and colleagues [9] found MMPs to be responsible for some of the repair inhibition by pro-inflammatory cytokines in a serum-containing porcine model. However, in our study most of the MMP-3 in the culture supernatant was in the pro-form, and it remains unclear to what extent this enzyme might have been involved in the present GAG release. But it is reasonable to believe that MMP-3 will be involved in the subsequent TNFα-dependent matrix degradation, as indicated by Wilson and colleagues [34]. TIMP-3, but not the other TIMPs, were able to inhibit the TNFα-induced GAG release and NITEGE production. This suggests that in the early three-day phase of meniscal proteoglycan degradation, aggrecanases must be involved. TIMPs are able to inhibit the active forms of almost all MMPs by binding to the C-terminal site of these enzymes [35]. However, TIMP-3 additionally inhibits ADAMTS-4 and -5 activity, whereas TIMP-1 and TIMP-2 have no effect on or even increase the activity of aggrecanases at concentrations of 1 μM or less [27,36-43]. According to our mRNA study, ADAMTS-4 might be one of the aggrecanases involved in TNFα-dependent proteoglycan degradation in bovine meniscal tissue, even though final evidence is still missing. This is supported by the fact that TIMP-3 inhibited, whereas TIMP-1 and -2 increased, the TNFα-dependent GAG release (in contrast to TIMP-3, TIMP-1 and -2 are known to stimulate the activity of ADAMTS-4 under certain conditions [43]). ADAMTS-4 mRNA has also been found in degenerated human menisci [44]. Therefore, it is likely that there might be similar effects in the human meniscus. Other studies showed that ADAMTS-5 mRNA was expressed next to ADAMTS-4 in osteoarthritic rabbit menisci [11]. Therefore, it is possible that both aggrecanases may play a role in the degradation of meniscal tissue. However, in the present investigation there was a basal meniscal mRNA expression of ADAMTS-4 in the bovine meniscus which increased with TNFα-treatment, whereas ADAMTS-5 mRNA expression was low or not detectable.

We were able to localize the NITEGE fragment in meniscal tissue by immunostaining in TNFα-treated explants, while it was almost non-detectable in control tissue. This is another strong indicator for aggrecanase involvement, according to many articular cartilage studies [14,15,45,46]. Additionally, TNFα-dependent NITEGE-formation could be blocked by TIMP-3, while TIMP-1 and -2 had no inhibitory effects (not shown). TIMP-3 is not a specific aggrecanase inhibitor. It has to be mentioned that it also regulates the activity of members of the membrane-bound ADAM-family, sheddases (a disintegrin and metalloproteinase: ADAM-10, -12 and -17; TACE [47-49]). The importance of these enzymes should therefore also be investigated in future studies.

We found a significant TNFα-dependent increase in meniscal NO production, which could be blocked completely by the common NO synthetase inhibitor L-NMMA. Although NO has been described as a meniscal product in several joint diseases and as an important mediator of meniscal tissue degradation in several studies [4,16-23], we did not see a stimulating influence of NO on the TNFα-induced GAG release or aggrecan cleavage. Our study suggests that NO is not involved in the early degradation of aggrecan in the meniscus. The slight but not significant increase in TNFα-induced GAG release after incubation with L-NMMA might reflect a protective function of endogenous NO in this context, as it has been shown previously by others [21].

## Conclusions

TNFα-treatment of meniscal tissue causes a reduced biosynthetic activity, release of GAG, degradation of aggrecan, and up-regulation of MMP-3 expression and aggrecanase activity. To our knowledge, this is the first report, showing that aggrecanase activity might be involved in the early TNFα-mediated aggrecanolysis in the meniscus. Inhibition of aggrecanase activity or TNFα-activity might therefore help to prevent meniscal destruction. TNFα also induces NO production, but it remains unknown what role NO might play in meniscal proteoglycan destruction because there is no evidence for a definite influence of endogenous NO on GAG release or aggrecan cleavage at the NITEGE site in this study.

## Abbreviations

ADAMTS: a disintegrin and metalloproteinase with thrombospondin motifs; ANOVA: analysis of variance; APMA: p-aminophenyl mercuric acetate; BSA: bovine serum albumin; C_T_: cycle of threshold; DMEM: Dulbecco's Modified Eagle's medium; GAPDH: glyceraldehyde-3-phosphate dehydrogenase; GAG: glycosaminoglycan; IL: interleukin; L-NMMA: NG-monomethyl-L-arginine.monoacetate; MMP: matrix metalloproteinase; NO: nitric oxide; OA: osteoarthritis; PBS: phosphate-buffered saline; RA: rheumatoid arthritis; RT-PCR: reverse transcription polymerase chain reaction; TIMP: tissue inhibitor of metalloproteinases; TNF: tumor necrosis factor.

## Competing interests

The authors declare that they have no competing interests.

## Authors' contributions

HV made the acquisition of data and part of the analysis of the data, and was also involved in drafting of the manuscript. AKL carried out the analysis and interpretation of mRNA data. RM made substantial contributions to conception and design of the study. MS revised the manuscript critically for important intellectual content. BK was involved in the conception and design of the study, analysis and interpretation of the data, and did most of the drafting of the manuscript. All authors read and approved the final manuscript.
